# Deciphering the origins of guanylate-binding proteins in mammals (Monotreme, Marsupials and Placentals)

**DOI:** 10.1186/s12915-025-02403-8

**Published:** 2025-10-01

**Authors:** João Vasco Côrte-Real, Ana Pinheiro, Jordan M. Sampson, Kimberly A. Morrissey, João Pedro Marques, Hanna-Mari Baldauf, Robert D. Miller, Joana Abrantes, Pedro José Esteves

**Affiliations:** 1 CIBIO-InBIO, Research Center in Biodiversity and Genetic Resources, University of Porto, 4485-661 Vairão, Portugal; 2 Faculty of Medicine, Max Von Pettenkofer Institute and Gene Center, National Reference Center for Retroviruses, LMU München, Munich, Germany; 3 Department of Biology, Faculty of Sciences, University of Porto, 4169-007 Porto, Portugal; 4 BIOPOLIS Program in Genomics, Biodiversity and Land Planning, CIBIO, Campus de Vairão, 4485-661 Vairão, Portugal; 5 Center for Evolutionary and Theoretical Immunology, Department of Biology, University of New Mexico, Albuquerque, NM USA; 6 CITS - Center of Investigation in Health Technologies, CESPU, 4585-116 Gandra, Portugal

**Keywords:** Evolution, Mammals, Marsupials, Monotremes, Multigene family, GBP

## Abstract

**Background:**

Guanylate-binding proteins (GBPs) belong to the large guanosine triphosphatases (GTPases) family and have specialised in host defence in vivo against a broad spectrum of invading pathogens. This ancient evolutionary group of genes was first studied in humans and rodents, but its evolution remained largely unknown for nearly 20 years. In recent years, more studies have emerged deepening the knowledge of *GBP* evolution in specific mammalian groups: Primates, *Tupaia*, Muroids (Rodents), Bats and Lagomorphs.

**Results:**

Here, we aimed to present a comprehensive analysis of mammals GBP evolution. Our phylogenetic analysis demonstrates that mammals’ GBPs share a common ancestor and that each major mammalian group has evolved a specific GBP repertoire. Two Monotreme GBP groups, *GBP8* and *GBP9*, cluster independently in the phylogenetic tree and do not share the synteny of the other mammalian GBP genes. The other two Monotreme GBP groups, *GBP1/2/3/5* and *GBP4/6/7*, are at the basal position of the main mammalian groups. Marsupials have two GBP groups, *Marsupial GBP1/2/3/5*, basal to *Placental GBP1/2/3/5*, and *Marsupial GBP4/6/7*, basal to *Placental GBP4/6/7*. *Marsupial GBP1/2/3/5* can be subdivided into three sub-groups, similarly to what is observed in the Placental GBPs, whereas *Marsupial GBP4/6/7* underwent several duplication events across species. We also examined the GBP tissue expression pattern in *Monodelphis domestica* and found that GBPs are ubiquitously expressed in most tissues, with some differences. Noteworthy was the presence of GBP transcripts in late foetal and newborn opossum tissues.

**Conclusions:**

The GBP genes revealed a distinct evolutionary pattern in each main mammalian group. Phylogenetic analysis shows that Monotremes and Marsupials have specific GBPs. Particularly intriguing is the presence of *GBP8* and *GBP9* only in Monotremes.

**Supplementary Information:**

The online version contains supplementary material available at 10.1186/s12915-025-02403-8.

## Background

All eukaryotes have an innate ability to resist invading pathogens due to a feature termed cell-autonomous immunity [1, 2]. One of the innate immune responses that present a robust first barrier against invading pathogens is the interferon (IFN)-mediated response [3, 4]. Increased production of type I IFN and type II IFN occurs upon stimulation of pattern recognition receptors (PRRs) by pathogen-associated molecular patterns (PAMPs) [4, 5]. Consequently, the expression of numerous IFN-stimulated genes (ISGs) increases [6]. Among the ISGs, the guanylate-binding proteins (GBPs) are among the most abundant and have specialised in host defence in vivo against a broad spectrum of invading pathogens and promote inflammasome signalling [1, 7–12]. GBPs belong to the large family of guanosine triphosphatases (GTPases) with a protein size between 65 and 72 kDa being predominantly localised in the cytoplasm [9, 13]. *GBP 1, 2* and *5* are associated with intracellular membranes (vesicle-like plasma membrane; perinuclear membrane; Golgi, respectively), due to a CaaX motif which allows isoprenylation and membrane anchoring [14–18].

GBPs are an ancient evolutionary group of genes, present in a broad range of eukaryotes (from plants to humans) [18, 19]. Olszewski and colleagues first started to unfold the evolution of GBP genes in humans and rodents [20], and it is accepted that GBPs emerged from a common ancestor [21]. In mammals, GBP genes are found in tandem in the genome and are typically located in a single gene cluster, except for muroids, which exhibit two GBP gene clusters [19, 20, 22, 23]. The evolutionary history of GBPs has been explored but remains unclear. In the last years, more studies have emerged deepening the knowledge of GBP evolution in specific mammalian groups. Primates have two exclusive GBP genes: *GBP3*, which seems to be exclusive to Simiiformes due to a duplication event of *GBP1*, and *GBP7*, which appears to be only present in Primates originating from *GBP4* [22]. In Rodents, the GBP genes show a pattern of gain and loss of genes, with *GBP1*, *GBP3* and *GBP7* not present in the Muroid genome. Yet, Muroids still have three orthologues to primates, *GBP2*, *GBP5* and *GBP6* [23]. Similarly, the Bats and Lagomorphs GBP genes also present a pattern of gain and loss of genes. In Bats, orthologues to *GBP1*, *GBP2* and *GBP4-6* were identified. *GBP2* has been lost in several Bat families whereas *GBP6* has duplicated into *GBP6a* and *GBP6b*, and *GBP1*, *GBP4* and *GBP6a* have suffered expansions [24]. In Lagomorphs, no *GBP6* orthologue was found, and species-specific expansions of *GBP4* and *5* have occurred [25]. Moreover, similar to Primates, *Tupaia* presents 5 *GBP* (*tGBP)* genes in a single cluster [26].

Multigene families have been characterised as groups of genes originating from a common ancestor and exhibiting similar DNA sequences and functions [27]. GBP DNA sequences share similarities, as such common amino acids are found and have been described to be structurally and biochemically related [7, 18, 19, 28]. Multigene families with functions related to the immune system have been proposed to follow the birth-and-death evolution process, which better explains why some genes are more phylogenetically related between species than within the same species compared to concerted evolution, as previously thought [29]. The birth-and-death model indicates that new genes are generated by duplication events, by tandem or block gene duplications [29]. These genes may diverge (remaining functional), acquire new functions or become pseudogenes if deleterious mutations occur, and can be either deleted or maintained in the genome [27]. Across several species, the GBP multigene family shares events of duplication, neofunctionalization, pseudogenization and deletion [19, 22–25, 27, 30]. Altogether, the GBP multigene family most likely follows the birth-and-death model of evolution.

Mammals can be divided into three major groups, the Monotremes, the Marsupials and the Placentals. These three groups shared an ancestor between ~ 163.7 and ~ 185.9 million years ago (Mya) [31]. The Monotremata can be divided into two families, Ornithorhynchidae, which contains the platypus (*Ornithorhynchus anatinus*), and Tachyglossidae, which includes the echidna (*Tachyglossus aculeatus).* The Australasian and American Marsupial Mammals, such as kangaroos and opossums, are the closest living relatives to Placental Mammals, having shared a common ancestor between ~ 130 and ~ 147 million years ago (Mya) [32, 33]. Marsupials are typically classified into two major superorders, the Australidelphia and the Ameridelphia [34, 35]. This division is partly based on differences in the ankle joints [36]. Additionally, it is estimated that the deepest split between the two superorders occurred ~ 80.6 Mya ago [37]. Australidelphia consists of five orders: Dasyuromorphia (carnivorous Marsupials and Marsupial mice), Peramelemorphia (bilbies and bandicoots), Notoryctemorphia (Marsupial moles), Diprotodontia (koalas, wombats, kangaroos, and possums), and the South American monotypic order Microbiotheria (monito del monte) [34, 38]. Ameridelphia consists of two orders: Didelphimorphia (opossums) and Paucituberculata (shrew opossums), mainly distributed in South America [34, 38, 39].

Here, we aimed to present a comprehensive phylogenetic analysis of mammalian GBPs, complemented by GBP tissue expression studies in *Monodelphis domestica *and* Ornithorhynchus anatinus*. To our knowledge, this is the first research concerning GBP evolution in the three groups of Mammals: Monotremes, Marsupials and Placentals. We have identified a distinct evolutionary pattern in each main mammal group.

## Results

### Sequence analysis and phylogeny

All sequences that were found annotated as GBPs in Marsupial and Monotreme genomes contained the most conserved GTP binding domains: GxxxxGK (S/T) and TVRD/TLRD, this last motif being characteristic of GBPs [12, 20, 40] (see Additional file 1: Table S1). Only three sequences did not present the GxxxxGK (S/T) domain, and these contained a partial deletion on the N-terminus. The motif TVRD/TLRD is present in most sequences, but in some genes, a minor amino acid variation is observed, where threonine (T) is exchanged by alanine (A), isoleucine (I) or valine (V). Additionally, the amino acid variation from valine (V) to leucine (L) is only observed in three sequences from the Australidelphia superorder (Additional file 1: Table S1). These exchanges are mainly observed in Monotremes, where all *GBP8* and *Monotreme GBP4/6/7* present one of these variations (Additional file 1: Table S1). The motif TVRD/TLRD is not present only in three sequences (Additional file 1: Table S1).

**Fig. 1:**
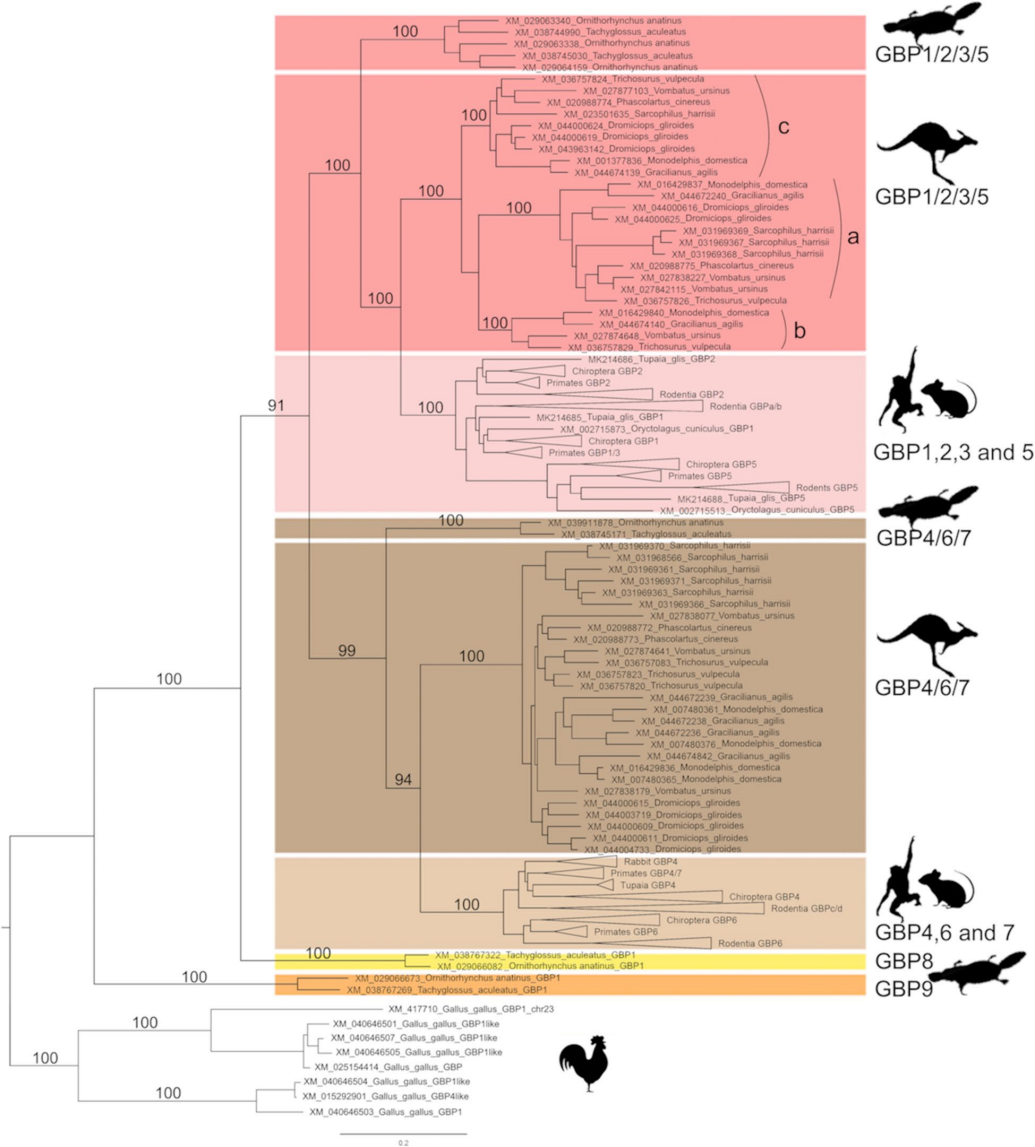
Phylogenetic tree of mammalian (Monotreme, Marsupials and Placentals) GBPgenes. The tree was obtained using the Randomised Axelerated Maximum Likelihood (RAxML) method [42, 43], the GTR + G + I model of nucleotide substitution and 1000 rapid bootstraps. The tree is represented with midpoint root. Bootstrap values are indicated near the most relevant branches. Scale bar refers to the inferred amount of change per site along branches

The 355 sequences obtained from the NCBI database were aligned allowing us to estimate the phylogenetic relationships. A Maximum Likelihood (ML) approach was followed using MEGA11 [41] and RAxML [42, 43] searches to obtain the phylogenetic tree. Both ML methods led to obtaining similar typologies, so only the RAxML tree is presented. The analysis of this phylogenetic tree revealed the presence of two main groups that include sequences of Placentals, Marsupials and Monotremes, one composed of sequences of *GBP1/2/3/5* and a second group consisting of sequences from *GBP4/6/7*. Two independent, Monotreme specific, groups are also observed. All Marsupial and Monotreme GBPs cluster within Marsupial and Monotreme specific groups and not among Placental Mammals’ sequences (Fig. 1). This suggests that all Marsupial and Monotreme GBPs previously identified as *GBP1* to *GBP7* are misclassified. The Marsupial sequences are divided into two main groups *Marsupial GBP1/2/3/5* (Fig. 1, in red) and *Marsupial GBP4/6/7* (Fig. 1, in brown). Both groups are well supported with bootstrap values of 100 (Fig. 1). The Monotreme sequences are divided into four main groups, all very well supported: the *Monotremes GBP1/2/3/5* and *Monotremes GBP4/6/7* (bootstrap values of 100, Fig. 1), which cluster with Marsupial and Placentals groups, and *Monotreme GBP8* and *Monotreme GBP9* (bootstrap values of 100, Fig. 1), which are independent of other groups and have basal positions in the ML tree.

*Marsupial GBP1/2/3/5* can be sub-divided into three sub-groups (Fig. 1): (i) *Marsupial GBP1/2/3/5a*, which is present across all Marsupials and has suffered duplications in three species belonging to separate orders, *Vombatus ursinus* (order Diprotodontia) and *Dromiciops gliroides* (order Microbiotheria) present two copies and *Sarcophilus harrisii* (order Dasyuromorphia) presents three copies in its genome (bootstrap 100; Fig. 1); (ii) *Marsupial GBP1/2/3/5b*, that encompasses one sequence for each of only four species from two orders, *Monodelphis domestica* and *Gracilinanus agilis* (order Didelphimorphia) and *Vombatus ursinus* and *Trichosurus vulpecula* (order Diprotodontia) (bootstrap 100, Fig. 1); and (*iii) Marsupial GBP1/2/3/5c* that is present across all Marsupials and all species present one copy except *Dromiciops gliroides* that has three copies in its genome (bootstrap 100; Fig. 1). The group *Monotreme GBP1/2/3/5* is well supported with 100 bootstrap value (Fig. 1; in red), with *Ornithorhynchus anatinus* having three copies and *Tachyglossus aculeatus* two copies. This group is located at the basal level of the main *GBP1/2/3/5* cluster (Fig. 1).

*Marsupial GBP4/6/7* is the second main cluster of GBPs in Marsupials and is highly supported with a bootstrap value of 100 (Fig. 1; in brown). In this group, we do not see a sub-division within the major cluster as seen in *Marsupial GBP1/2/3/5*. Instead, sequences are clustered according to species and their phylogenetic relationship (Fig. 1). Duplication events of Marsupial *GBP4/6/7* occurred across the seven species analysed; the genome of *Sarcophilus harrisii* encodes a total of six copies, and *Dromiciops gliroides* presents five copies (Fig. 1). In the order Didelphimorphia, both species, *Monodelphis domestica* and *Gracilinanus agilis*, encode four copies (Fig. 1). *Vombatus ursinus* and *Trichosurus vulpecula* present three copies and *Phascolarctos cinereus* genome encodes two copies (Fig. 1). The *Monotreme GBP4/6/7* encodes a single copy in each of the two species analysed and is a well-supported group with a bootstrap value of 100 (Fig. 1; in blue).

**Fig. 2:**
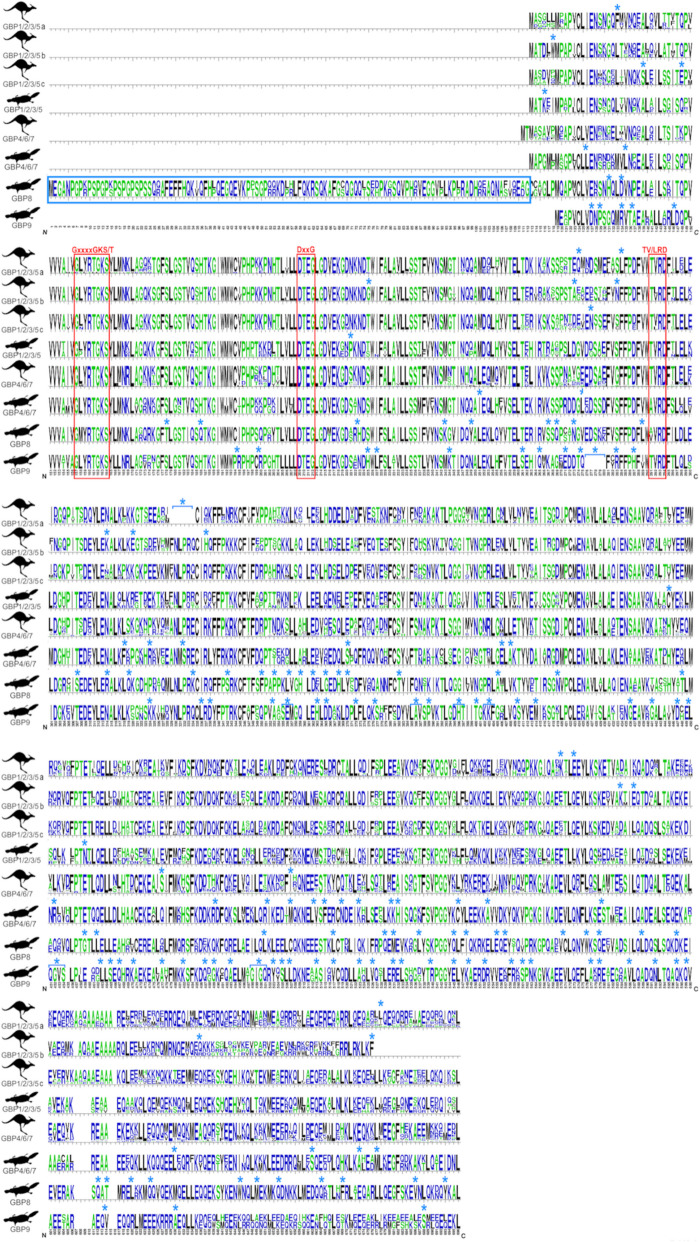
Marsupials and Monotreme GBPamino acid sequences diversity comparison. Alignments of the Monotreme *GBP8, 9, 4/6/7* and *1/2/3/5* and Marsupials *GBP4/6/7*, *1/2/3/5a*, *1/2/3/5b* and *1/2/3/5c* amino acid sequences were used to create the sequences’ logo graphical representations, using the WebLogo programme [47]. Amino acid residues that differentiate each GBP are identified with an asterisk above the sequence. The *GBP8* extra 106–112 amino acids in the N-terminus are in a blue box. Vertical red boxes identify the conserved GTP binding motifs, P-loop (GxxxxGKS/T), switch II (DxxG) and T(V/L)RD motif, involved in GTP binding/orientation and GTP hydrolysis [44, 49–51]. The last 144 amino acids of the C-terminal, where the CaaX box is located, are not shown. Slashed vertical rectangle indicates a gap resulting from the alignment with *G. gallus* and/or Placentals’ GBPs

The two independent Monotreme groups, *Monotreme GBP8* (Fig. 1; in yellow) and *Monotreme GBP9* (Fig. 1; in orange), encode a single-copy gene for each one of the two Monotreme species analysed. *Monotreme GBP8* has an extra 106–112 amino acids in the N-terminus (Fig. 2) and at the C-terminus a CaaX motif. The presence of this CaaX motif indicates that this motif was present in the most recent common ancestor of Monotremes and Marsupials, between ~ 163.7 and ~ 185.9 Mya (31). *Monotreme GBP9* is basal to all other GBPs.

Given the obtained results, we propose a new classification following the inferred ML tree (Additional file 1: Table S1), where Marsupials present only two main groups of GBPs, *Marsupial GBP1/2/3/5 *and* Marsupial GBP4/6/7*, while Monotremes present four groups, *Monotreme GBP1/2/3/5, Monotreme GBP4/6/7, Monotreme GBP8 *and* Monotreme GBP9*. Characteristic amino acids further support the new Monotreme and Marsupial GBP groups (Fig. 2; see Additional file 2: Data 1 for sequence alignment).

### Synteny analysis

**Fig. 3:**
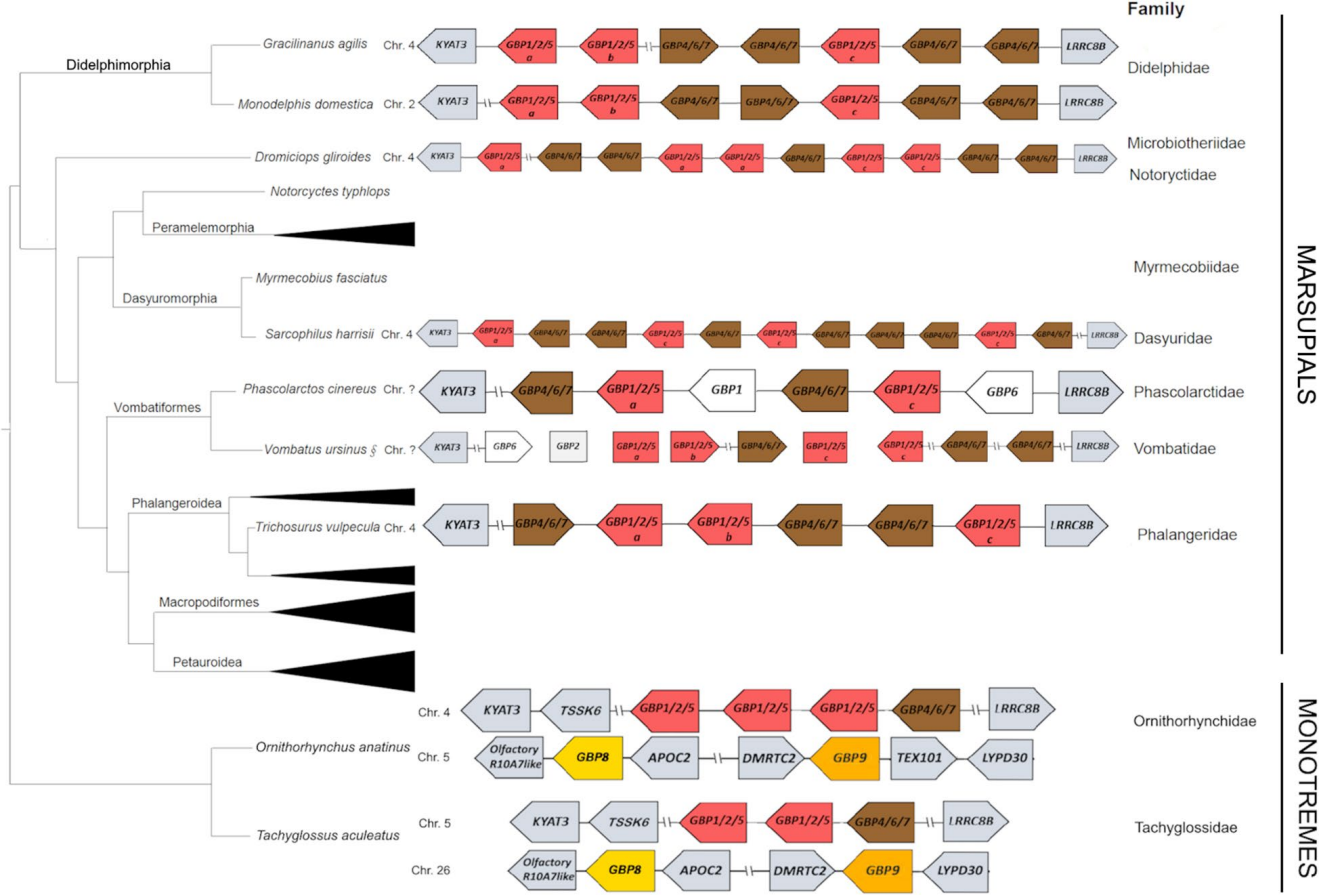
GBP gene family synteny in Monotreme and Marsupials. Organisation of the GBP gene family in the studied species according to genomes available in NCBI (www.ncbi.nlm.nih.org). The diagram is not drawn to scale. Arrows represent transcription orientation. The phylogenetic tree of these groups is shown to the left. Chromosomes are indicated when information is available. Double slashes indicate a greater gap/presence of other genetic elements between the represented genes

Synteny of GBP genes in Marsupials shows that GBPs are flanked by *KYAT3* and *LRRC8B*, (Fig. 3). Didelphimorphia, *Dromiciops gliroides* and *Sarcophilus harrisii* appear to possess *GBP1/2/3/5* genes adjacent to *KYAT3* and *GBP4/6/7* genes adjacent to *LRRC8B* (Fig. 3), following the same patterns observed in primates. However, *Phascolarctos cinereus* and *Trichosurus vulpecula harrisii* have *GBP4/6/7* genes adjacent to *KYAT3* and *GBP1/2/3/5* genes adjacent to *LRRC8B* (Fig. 3). The synteny of GBP genes could not be fully assessed for *Vombatus ursinus*, as this region is still fragmented. However, a cluster of GBPs is located adjacent to *LRRC8B* and a GBP pseudogene is adjacent to KYAT3 (Fig. 3). In Monotremes, a GBP cluster containing genes from *GBP1/2/3/5* and *GBP4/6/7*, similar to the Marsupials and Placentals clusters, is present, flanked by *TSSK6* and *KYAT3* on the *GBP1/2/3/5* genes’ extreme and by *LRRC8B* adjacent to *GBP4/6/7* genes (Fig. 3).

Monotremes *GBP8* and *GBP9* are located on a different chromosome and are separated by nearly 1000 kbp. In both species, flanking genes around *GBP8* are *Olfactory receptor 10A7-like* (*OR10A7l*) and *APOC2*, and *DMRTC2* and *LYPD3* are flanking *GBP9* (Fig. 3). These four flanking genes are present in the Marsupials and Placentals’ genomes, also the four located on the same chromosome, but with much greater distances in between and not always in the same relative positions. This shows that this is a dynamic region that has suffered chromosomal rearrangements. The genes surrounding each one of the four reference genes, *OR10A7l* and *APOC2*, *DMRTC2* and *LYPD3*, are mostly conserved, except for the Marsupial genomes, which show a greater instability. No GBP partial sequences, pseudogenes, or traces of deletions were found in these regions for non-Monotreme mammals (Additional file 3: Figure S1). To investigate the *GBP8* and *GBP9* origin, we also looked at the *Gallus gallus GBP* synteny. Of the eight genes identified as GBP in the *G. gallus* genome, seven are located in tandem, in chromosome 12, flanked by *PHF7-like* and *DCAF* genes. The eighth *G. gallus GBP* gene (accession number: XM_417710) is located on chromosome 23, flanked by *CITED4* and *STX12*. *OR10A7l*, *APOC2*, *DMRTC2* and *LYPD3* genes were not identified in the *G. gallus* genome. Interestingly, this single *G. gallus GBP* has an unusually long N-terminus, with 410 extra amino acids. Despite the coincident resemblances between Monotremes’ *GBP8* and this eighth *G. gallus GBP*, the exceptionally long N-terminus and location as a single gene in a different chromosome of the GBP cluster, neither the synteny nor the results obtained in the phylogenetic tree indicate that this *G. gallus GBP* has originated Monotremes’ *GBP8*.

### Evolutionary model

Considering the obtained phylogeny and the synteny analysis, we proceeded to test three different scenarios for the origin of Monotreme *GBP8* and *GBP9*. To do so, constraint analyses were used to evaluate the phylogenetic placement of *GBP8* and *GBP9*. We compared three alternative topologies:

- Hypothesis A—the unconstrained ML tree, where *GBP9* and *GBP8* are progressively nested within the *GBP1–7* clade, suggests that the ancestral GBP duplicated and, after chromosomal rearrangements, gave rise to *GBP8* and *GBP9*, which were retained in Monotremes but lost in Marsupials and Placentals.
- Hypothesis B—*GBP8* and *GBP9* are nested within the broader *GBP1–7* clade—which implies that the ancestral *GBP* duplicated only in the Monotreme lineage and, after chromosomal rearrangements, gave rise to *GBP8* and *GBP9*.
- Hypothesis C (*GBP8* and *GBP9* form a sister group to *GBP1–7*)—the ancestral GBP duplicated into *GBP8/9* and *GBP1/2/3/4/5/6/7* ancestors, which gave rise to the GBP diversity observed today.

Statistical tests comparing log-likelihoods (Shimodaira–Hasegawa (SH) and Approximately Unbiased (AU)) did not reject any of the topologies (all *p*-values > 0.05, Additional file 1: Table S2), indicating that all three hypotheses are compatible with the data.

### Expression pattern of GBP in laboratory opossum and platypus

**Fig. 4:**
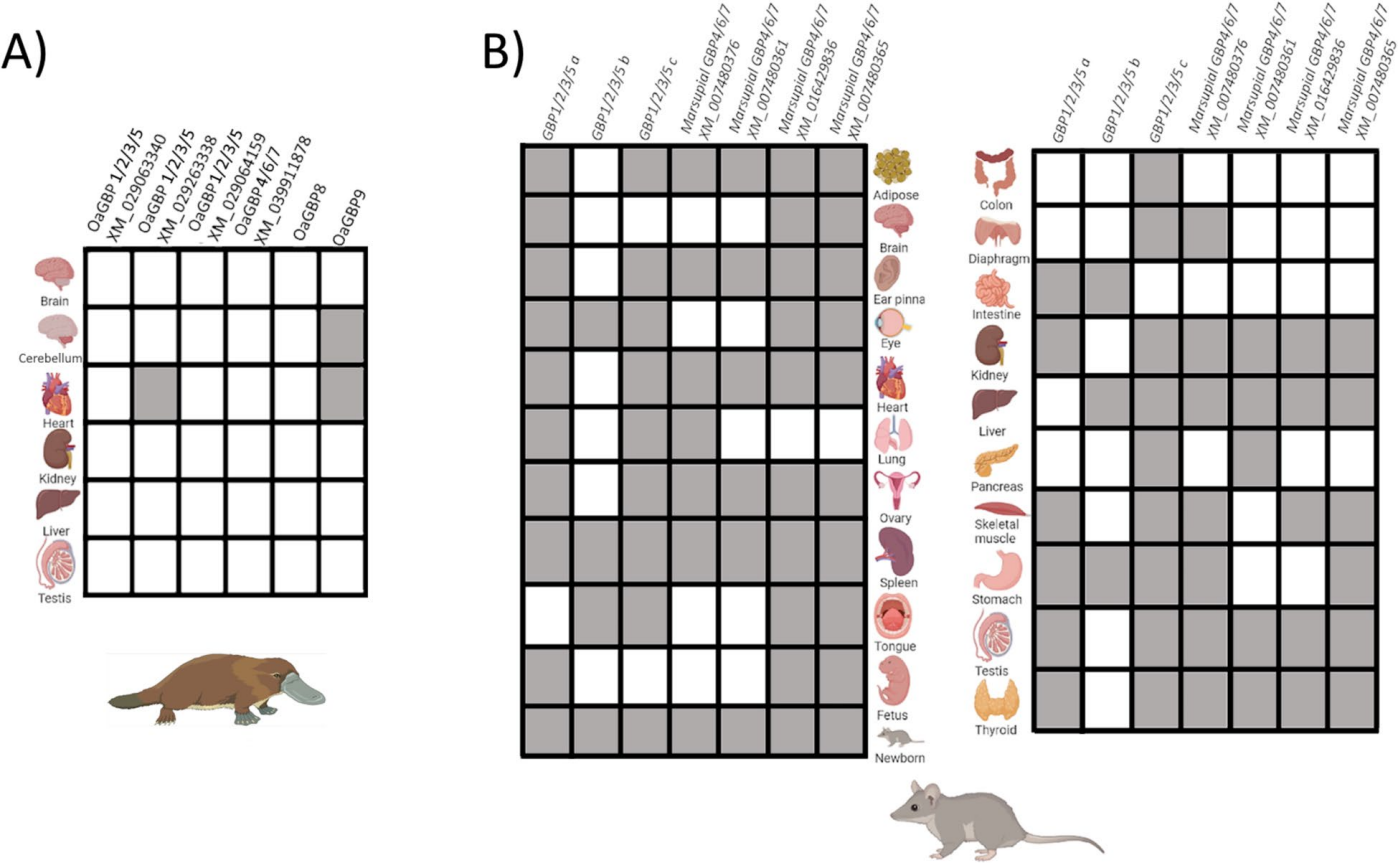
Expression pattern of** GBP **in laboratory Opossum and Platypus.** A** Differential mRNA tissue expression for *Monodelphis domestica GBPs* was determined using an available opossum dataset transcriptomes [68] (NCBI accession number PRJNA200320). **B** Tissue distribution of the platypus GBP were determined using a publicly available transcriptome dataset (accession number PRJNA143627) [60]. Grey colour means present, white means absent

Next, we investigated the expression pattern of GBPs in the grey, short-tailed opossum, *Monodelphis domestica*, and platypus, *Ornithorhyncus anatinus*. Twenty-one opossum tissues were analysed, Marsupial GBPs were ubiquitously expressed in all tissues (Fig. 4). The spleen was the only tissue where all GBPs were detected in the transcriptome, followed by adipose tissue, ear pinna, heart, kidney, ovary, testis and thyroid where all GBPs were detected except for *Marsupial GBP1/2/3/5b* (Fig. 4). The tissue with the least *GBP* expression was the colon where only *Marsupial GBP1/2/3/5c* was detectable (Fig. 4). Diaphragm, intestine and pancreas expressed only two different GBPs. In the intestine, no *Marsupial GBP4/6/7* was detected, only *GBP1/2/3/5a* and *c* were found. Diaphragm and pancreas expressed *GBP1/2/3/5c* and a *GBP4/6/7* gene, *Marsupial GBP4/6/7* (XM_007480376) and *Marsupial GBP4/6/7* (XM_007480361), respectively. *Marsupial GBP1/2/5c* was the most expressed GBP, detected in 18 tissues, followed by *Marsupial GBP1/2/5a* and *Marsupial GBP4/6/7* (XM_007480365), with both being expressed in 16 tissues, then *Marsupial GBP4/6/7* (XM_016429836) was detected in 15 different tissues. The least expressed GBP was *Marsupial GBP1/2/5b* which was only detected in seven tissues (Fig. 4). In the foetus, three GBPs were detected, *GBP1/2/3/5a*, *Marsupial GBP4/6/7* (XM_016429836) and *Marsupial GBP4/6/7* (XM_007480365), whereas in the newborn all GBPs were detected (Fig. 4).

Six platypus tissues were evaluated, four of which, brain, heart, kidney and testes, did not show any transcription of the Monotreme GBPs (Fig. 4)*.* The remaining two tissues, cerebellum and heart, did show transcription of two of the Monotreme *GBP: OaGBP1/2/3/5* and *OaGBP9* (Fig. 4). *OaGBP9* was found in both the heart and cerebellum and *OaGBP1/2/3/5* was found only in the heart (Fig. 4).

Overall, *Marsupial GBPs* were constitutively expressed across the different tissues in a similar pattern to other Placental species, but *Monotreme GBPs* were only found expressed in two tissues, in striking contrast. However, there were limitations, such as the lack of information on transcript and protein expression levels in the available *Monodelphis domestica* tissues, as well as the limited number of tissues available for *Ornithorhynchus anatinus*. This lack of data makes comparisons between *Monodelphis domestica, Ornithorhyncus anatinus* and other species, such as humans, mice, *Oryctolagus cuniculus* and *Tupaia,* difficult.

## Discussion

Guanylate-binding proteins have important roles in immunity, being involved in the cell-autonomous innate immune response against bacterial, parasitic and viral infections [28]. Despite their relevance, evidenced by their presence in a broad range of eukaryotes (from plants to humans) [18, 19], the evolution of GBPs has been mainly addressed in humans [18]. Lately, we have expanded the knowledge on GBP’s evolution to several mammalian groups such as Primates [22], Muroids (Rodentia) [23], Lagomorphs [25] and Chiroptera [24]. These studies disclosed a pattern of evolution with gain and loss events in these groups leading to a different GBP gene repertoire in each group. With the present work, we sought to expand the knowledge of GBPs to the most ancient mammalian groups, the Monotremes and the Marsupials, and to obtain an evolutionary model for mammalian GBPs.

Searching public databases, we obtained 62 sequences annotated as Monotreme and Marsupial GBPs. The presence of conserved GTP binding domains GxxxxGK (S/T) and TVRD/TLRD [20] in all these GBPs indicates that these genes belong to the GBP family. Unlike other GTPases, which contain an (N/T)(K/Q)xD motif, GBPs changed to a unique TVRD/TLRD motif for GTP binding, which identifies these proteins [12]. In the TVRD/TLRD motif, the conservative exchange of threonine for hydrophobic amino acids (A/I/V) has already been observed in Rodents and Lagomorphs and has been present since the origin of Mammals, thus, it most likely presents a minor functional consequence [44]. Interestingly, in the mouse and Primates, the TLRD motif is associated with *GBP1*, *2* and *5* in addition to the CaaX motif while the TVRD is linked with *GBP4*, *6* and *7* [20]. However, this pattern is not observed in Marsupials and Monotremes, which indicates that this association between the TLRD and CaaX motifs only emerged in the Placentals ancestor between 66 and 102 mya [32, 45–48]. The ancestral motif most likely was TVRD, since in both Marsupials and Monotremes it was the most common motif found in all GBPs (Additional file 1: Table S1). Analysing the *Gallus gallus GBP* sequences used as outgroup in this study, we verified that these have a TVRD/VVRD motif (see Additional file 2: Data 1 for sequence alignment), corroborating that the TVRD was the mammal’s ancestral motif.

Our phylogenetic analysis demonstrates that all mammals’ GBPs share a common ancestor. Still, each major mammalian group has evolved a specific subset of GBP genes, as shown by the existence of well-supported GBP clusters for each mammalian group in our ML tree (Fig. 1). This is in line with our previous studies on several placental mammal groups [22–25]. Monotremes present four GBP clusters, two of which are basal to all other mammalian GBPs, and Marsupials present two GBP clusters. As such, we suggest a new nomenclature for these groups (see Additional file 1: Table S1). All groups are well supported with high bootstrap values and present characteristic amino acids. Characteristic amino acids further support the newly proposed groups (Fig. 2). Analysing the GBPs of ancestral mammals, this study confirms that *GBP3* and *GBP7* clusters are only present in primates [22].

### GBP evolutionary models in mammals

**Fig. 5:**
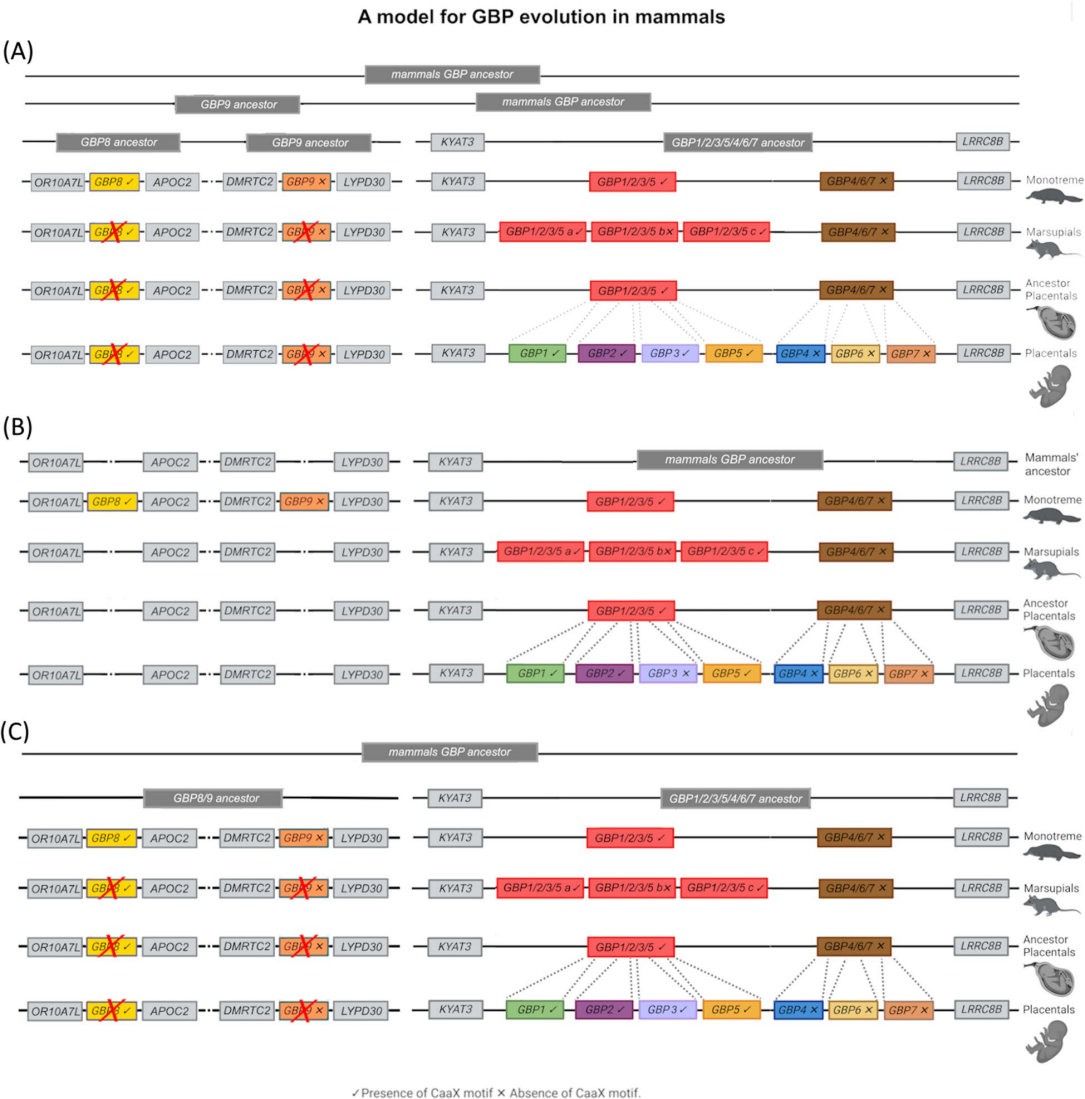
Schematic representations of the possible evolution of GBPs in mammals. Integrating our ML and synteny analysis results, we tested three putative evolutionary models that could explain the origin and evolution of GBPs in mammals. **A** Hypothesis A—the mammals’ GBP ancestor sequentially duplicated, originating the *GBP8*, *GBP9* and *GBP1/2/3/5 *and* GBP4/6/7*. Monotremes maintained these four genes, but *GBP8* and *GBP9* were lost in Marsupials and Placentals. **B** Hypothesis B—the mammals’ GBP ancestor first duplicated into the *GBP1/2/3/5* and *GBP4/6/7* ancestors. In Monotremes, the *GBP1/2/3/5* and *GBP4/6/7* were maintained, and two new genes emerged, *GBP8* and *GBP9*, in a different chromosomal location. **C** Hypothesis C**—**the GBP ancestor duplicated into the *GBP8/9* and *GBP1/2/3/5/4/6/7* ancestors, which duplicated originating *GBP8*, *GBP9*, *GBP1/2/3/5 *and* GBP4/6/7*. Monotremes maintained these four genes, but *GBP8* and *GBP9* were lost in Marsupials and Placentals

Based on previous analysis in primates, we assumed that the GBP gene cluster arose from a common ancestor [20]. Moreover, it has been hypothesised that after the first duplication, one gene gave rise to modern GBPs 1, 2, 3 and 5, and the other to GBPs 4, 6 and 7 [20]. Based on our ML analysis, we hypothesised that the *GBP* ancestor first duplicated into the *GBP8*, *GBP9* and *GBP1/2/3/5/4/6/7* ancestors (Hypothesis A), which then duplicated and originated four genes: *GBP9*, *GBP8* and *GBP1/2/3/5* and *GBP4/6/7* (Fig. 5A). Monotremes maintained these four genes, but Marsupials and Placentals lost *GBP9* and *GBP8*. Considering the analysis of the mammalian GBP’s syntenic regions, in which no partial sequences, pseudogenes or traces of deletions of *GBP8* and *9* were found in the syntenic regions of Marsupials and Placentals, we alternatively hypothesised that the mammals’ GBP ancestor first duplicated into the *GBP1/2/3/5* and *GBP4/6/7* ancestors. In Monotremes, the *GBP1/2/3/5* and *GBP4/6/7* were maintained, and two new genes emerged, *GBP8* and *GBP9*, in a different chromosomal location (Hypothesis B; Fig. 5B). A third hypothesis would be that the GBP ancestor duplicated into the *GBP8/9* and *GBP1/2/3/5/4/6/7* ancestors (Hypothesis C; Fig. 5C). Our ML tree suggests that *GBP8* and *GBP9* form distinct clades that were present in the ancestral genome. In this scenario, we would expect to find traces of *GBP8* and *GBP9* in non-Monotremes after their loss in these groups. Our analysis of the mammals’ syntenic region corresponding to the Monotreme *GBP8* and *GBP9* regions failed to reveal remnants of these genes; it also showed that these are very dynamic regions, having undergone chromosomal rearrangements that may have caused the absence of such remnants. Constraint-based tests did not reject our ML tree (Hypothesis A) or alternative hypotheses in which *GBP9* and *GBP8* are nested within the *GBP1–7* lineage (Hypothesis B) or form a sister clade to *GBP1–7* (Hypothesis C). These results suggest that the current dataset does not have enough phylogenetic signal to conclusively resolve the placement of *GBP8* and *GBP9* (Additional file 1: Table S2). Additional data, broader taxonomic sampling, or complementary genomic context (e.g. synteny or functional divergence) may be required to clarify their evolutionary history.

After the Marsupials and Placentals split, *GBP1/2/3/5* gave rise to *Marsupials GBP1/2/3/5a, GBP1/2/3/5b* and *GBP1/2/3/5c* and *Placentals GBP1*, *2* and *5*, with *GBP3* arising only in Primates as described elsewhere [22]*.* A similar pattern is observed for the *GBP4/6/7* gene. For the *Marsupials GBP4/6/7* group, despite having undergone several duplication processes, the division of this group, as placentals *GBP4*, *6* and *7*, is not possible since no clear gene clusters appear. Instead, it clusters by species, indicating that all *Marsupial GBP4/6/7* are in the same family of genes. In Placentals, this group differentiated into two genes, *GBP4* and *6*, with *GBP7* duplicating from *GBP4* in primates as previously described [22]. Interestingly, *Monotreme GBP8* has a carboxy-terminal CaaX motif, comparable to *GBP1*, *2* and *5* (Fig. 5). This suggests the CaaX motif might have been present in the mammals’ GBP ancestor gene and was then lost in three independent lineages, *Monotremes GBP9*,* GBP4/6/7* and *Marsupial GBP1/2/5b*. Since *Marsupial GBP1/2/5b* is present in both Marsupial superorders, it seems that the loss of the CaaX motif occurred in the common ancestor and that the gene might have been deleted from some Marsupial species. *Marsupial GBP1/2/5b* was found to be the least expressed gene in *Monodelphis domestica*. Altogether, these findings suggest that *Marsupial GBP1/2/3/5b* could be a gene in the process of being deleted or pseudogenized from the genome of all Marsupials.

The model of birth-and-death evolution was proposed [29] to explain the evolution of multigene families that did not fit the concerted evolution model. According to this model, when duplication events give rise to new copies of a gene, and these persist in the genome for long periods of time, divergence will lead to different fates. Some copies may diverge and remain functional, while others will diverge more and split functions (subfunctionalization), acquire new functions (neofunctionalization) or become pseudogenes if deleterious mutations occur, which can either be deleted or maintained in the genome [27, 29]. As previous studies have demonstrated, the evolutionary history of the GBP multigene family in mammals is complex, with duplications, deletions and neofunctionalization in several groups [22–25]. The *GBP3* gene is a Simiiformes-specific GBP, emerged through a duplication of *GBP1* [22] and gained a new function: regulation of caspase-4 activation [30]. Other genes have emerged through duplication, such as Primates *GBP7* [22], Rodents *GBPa, b, c* and *d* [23], Leporidae *GBP5* and *GBP4* multiple copies [25], and Bats *GBP6a* and *6b* [24], though the function of these new genes is yet to be studied. In contrast, genes have been deleted from some mammalian lineages, such as *GBP4* and *GBP5* which have been deleted from the genomes of Old-World monkeys, *GBP6* has been deleted from the Lagomorphs genome, *GBP1* which is not present in the Muroids genome, and *GBP2* has been lost in several bat families [22–25]. Given this context, it is not entirely surprising to find new genes in the phylogenetically more basal mammals. Given the divergence between *GBP8* and *9* and the other GBPs, it is possible that these two genes have acquired new functions.

In each mammal group, the evolution of GBPs presents a pattern of gene gain and loss and the mechanisms that regulate this are not yet understood. It is possible that evolutionary pressures due to the environment and invading pathogens could dictate the birth and death of genes. Despite this, GBPs share common features and their functions seem to be conserved in some mammalian species (i.e. human, mice, *Tupaia* and *Oryctolagus cuniculus*) [18, 25]. The N-terminal GTPase domain is the most conserved domain and includes five motifs, P-loop (GxxxxGKS/T), switch I (T), switch II (DxxG), T(V/L)RD motif and the guanine cap, involved in GTP binding/orientation and GTP hydrolysis [44, 49–51). These can be found in several Mammal species (human, pig, mouse, *Tupaia* and *O. cuniculus*) and can also be found in Marsupials and Monotremes, hinting at shared common functions. Additionally, *GBP1*, *2* and *5* present the CaaX motif which allows isoprenylation and consequently target GBPs to intracellular membranes [52]. The CaaX can be found also in *Monotreme GBP1/2/3/5* and *GBP8* and *Marsupial GBP1/2/3/5 a* and *b*, indicating the ability of these proteins to anchor to membranes and enabling the destruction of pathogen-containing vacuoles (mainly bacteria) [9, 18]. Moreover, *Danio rerio GBP3* and *4* have been described as having physical and functional interactions with inflammasome similar to human GBPs [3].

GBPs are ubiquitously expressed in humans, mice, *Tupaia* and *O. cuniculus* [3, 25, 26], the same is observed in Opossum. It was demonstrated that the expression of GBPs is increased upon stimulation by IFN (human, mice, *Tupaia* and *O. cuniculus*) indicating a function related to the cell-autonomous innate immune response by providing defence against a broad range of invading pathogens. Indeed, it was described that mouse and human GBPs offered protection against bacteria (*L. monocytogenes* and *M. bovis*) and protozoan pathogen *T. gondii* [53]. Additionally, GBPs promote antiviral activities against a broad range of viruses interfering with viral replication through different mechanisms (recently reviewed [18, 53]). It was also observed that *GBP5* from *O. cuniculus* was able to inhibit furin activity, similarly to *hGBP2/5* [25, 54], suggesting that *ocGBP5* could interfere with the proper cleavage of virus glycoproteins (HIV, influenza A, zika virus and measles).

It seems that GBPs have consistent features across several species; however, newly formed genes could gain new functions, as observed in *hGBP3* [30]. This suggests that each *GBP* could gain a specific function, and it would be interesting to investigate the functions of *GBPs 8* and *9* in Monotreme since they are not found in any other mammal.

Overall, it seems that the evolution of GBPs does not influence the functions related to innate immunity (i.e. GTPase activity and IFN inducibility), remaining conserved across different families from fish to mammals, suggesting that Marsupial and Monotreme GBPs could have a role in the innate immunity. However, we cannot discard the possibility of some GBPs presenting specific functions to Marsupials and Monotremes. As such it is paramount to perform a functional characterisation of these proteins to further understand their function.

### GBP expression in laboratory opossum and platypus

Expression profile of GBPs has been described in humans, mouse, European rabbit and *Tupaia* [20, 25, 26, 40, 55]. We examined the expression profile of 19 adult tissues, pooled newborn opossum, and pooled foetal tissues at terminal gestation in *M. domestica*. We observed that GBPs are ubiquitously expressed in 19 different adult tissues, similarly to human, mouse, European rabbit and *Tupaia* [20, 25, 26, 40, 55]. However, no comparison regarding each GBP group can be made. It should be noted that this publicly available *M. domestica* transcriptome dataset used here was generated from normalised total RNA libraries generated as part of the opossum genome project [56]. Normalised libraries allow the discrimination between the presence or absence of any particular transcript; however, relative or quantitative information on the level of transcription is not available from this dataset.

Opossums, like all Marsupials, are born highly altricial, at a state similar to a foetal human or mouse [57]. Initiation of T cell development in the opossum can be first detected in the last day of gestation [58] and mature T cells are not detected until postnatal day 2 [59]. Likewise, B cell development occurs entirely postnatally with mature B cells undetectable until the end of the first postnatal week [60]. The opossum adaptive immune system, therefore, develops postnatally. The same is likely true for all Marsupials and it has long been established that newborn Marsupials are born “immune-incompetent” and fail to mount significant adaptive immune responses until they are often 2 to 3 weeks old [61, 62]. The nature of their short gestation periods followed by postnatal “foetal-like” development clearly leaves the newborn Marsupial highly dependent on both maternal immunity as well as their own innate immune system. It is noteworthy, therefore, that we found GBP transcripts in late foetal opossum tissues. The diversity of transcripts was particularly broad as well in newborn opossums. These results are consistent with GBP being available to the newborn opossum at birth to contribute to the generation of innate-like immune responses.

In Platypus, the expression of GBPs in the six analysed tissues was lower than that observed for Placentals and Marsupials. A study of individual gene expression shifts in six tissues for mammalian species (including primates, mouse, opossum and platypus) showed that the Platypus has the highest number of gene expression switches in most organs [60], which can help to explain the observed pattern. Furthermore, shifts in gene expression are often larger after gene duplication, and accompanied by accelerated or decelerated rates of protein evolution, supporting the idea that gene duplication tends to free genes up for regulatory or structural functional divergence, and sometimes both [61]. These features could be related to the birth and death model evolution that drives the evolutionary patterns of the GBP genes, originating subfunctionalisation or neofunctionalisation.

## Conclusions

In this study, we collected sequences annotated as Monotreme and Marsupial GBPs in public databases. The presence of conserved motifs, as the GTP binding domains GxxxxGK (S/T) and TVRD/TLRD, indicates that these genes belong to the GBP family. However, this study’s results show that the nomenclature of these genes should be revised as they are incorrectly annotated as orthologs to placental mammals’ GBP genes. As genome annotation continues, and new genomes of higher quality are generated, it is very relevant to improve the gene annotation by identifying new genes and revising some previously misannotated genes, for which evolutionary analysis, especially in multi-gene families, should be a necessary step. The results of this study show that several evolutionary processes occurred in the GBP gene family, such as gene deletions and duplications, and possible pseudogenisation. These data are in accordance with the birth-and-death model of evolution, already attributed to members of this multigene family. In line with what was previously observed in the Placental groups, the Monotreme and Marsupials have specific GBP gene repertoires, suggesting that these genes have been evolving under host–pathogen co-evolution. Our phylogenetic and synteny analyses show, surprisingly, that Monotremes have two GBPs, *GBP8* and *GBP9*, that are not related to the remaining mammals’ GBP genes. This work shows that the placentals *GBP 1–7* have a common ancestor dating back to the oldest mammalian ancestor, which has undergone lineage-specific duplications giving rise to the extant mammalian GBPs. The GBP tissue expression pattern in *Monodelphis domestica* showed the presence of GBP transcripts in late foetal and newborn opossum tissues which is consistent with GBP being available to the newborn opossum at birth to contribute to the generation of innate-like immune responses. Functional studies should be carried out to fully understand the role of Monotreme and Marsupial GBPs, with particular emphasis in the Monotreme unique *GBP8* and *GBP9.*

## Methods

### Phylogenetic analysis

We retrieved 355 publicly available gene sequences annotated as GBP (see Additional file 1: Table S1 for accession numbers) from 5 different species of Marsupials, representing five Marsupial orders [51 sequences), 2 species of Monotreme order (11 sequences), 6 species of primates (including *Homo sapiens*; 41 sequences [22]), 12 species of rodents (123 sequences [23]), 19 species of bats (113 sequences [24]), European rabbit (*Oryctolagus cuniculus*, 4 sequences [25]), *Tupaia glis* [5 sequences [26]) and *Gallus gallus* was used as an outgroup to root the tree (8 sequences). The Marsupial and Monotreme species included in our study, have a high-quality genome assembly, with high genome coverage (lowest coverage of 57% for Phascolarctus cinereus), and most genomes present no gaps (only three species present some gaps, with the total ungapped length between 92 and 97%, *Gracilinanus agilis*, *Ornithorhynchus anatinus* and *Trichosurus vulpecula*). BLAST (Basic Local Alignment Search Tool) searches against each species-specific whole-genome were carried out, using the identified sequences as queries, to ensure that all *GBP* sequences were included, from the Marsupial and Monotreme species analysed. No new GBPs were identified, further to those annotated. Sequences that did not encode a functional protein and presented partial mRNA sequence were excluded from the analysis (see Additional File1: Table S1 for accession numbers). Sequence alignment was performed in BioEdit [63] using Clustal W method [64] followed by visual inspection. The last ~ 430 nucleotides were excluded from the alignment; the high variability in this region makes it very difficult to obtain a good alignment of this region (see Additional File 4: Data2 for the nucleotide alignment). We then screened the dataset for the presence of gene conversion/recombination using GARD [65]. No gene conversion/recombination events were detected. Phylogenetic relationships were estimated using a Maximum Likelihood (ML) approach in MEGA 11 [66] and RAxML (Randomised Axelerated Maximum Likelihood) v8.2.12 [42, 43]. The phylogenetic tree was constructed using the GTR + G + I model of nucleotide substitution, determined to be the best fitting model to our dataset by the Model Selection option in MEGA 11 [66]. Node support was determined from 1000 bootstrap replicates.

To evaluate alternative evolutionary hypotheses for the origin of GBP8 and GBP9, we performed phylogenetic constraint analyses using IQ-TREE v3.0.1 [67]. Three topologies were tested:

(i) A topology in which GBP9 and GBP8 are progressively nested within the GBP1–7 clade (Hypothesis A, corresponding to our unconstrained ML tree) - ((GBP9,((GBP8), (GBP1,GBP2,GBP3,GBP4,GBP5,GBP6,GBP7))),Outgroup);

(ii) A topology in which GBP8 and GBP9 are nested within the broader GBP1–7 clade (Hypothesis B) - ((GBP1,GBP2,GBP3,GBP4,GBP5,GBP6,GBP7,GBP8,GBP9),Outgroup); and

(iii) A topology in which GBP8 and GBP9 form a sister clade to GBP1–7 (Hypothesis C) - (((GBP8,GBP9),(GBP1,GBP2,GBP3,GBP4,GBP5,GBP6,GBP7)),Outgroup).

In all cases, outgroup taxa were constrained to form a basal monophyletic clade. The log-likelihood scores of the three topologies were compared using the SH [68] and AU tests [69] with 10,000 replicates.

### Genomic synteny analysis

The GBP syntenic positions and transcription orientations were inferred by visualising the genome of Placentals, Marsupials and Monotremes in publicly available databases: NCBI (https://www.ncbi.nlm.nih.gov/genome/gdv/) and Ensembl (https://www.ensembl.org/index.html).

### Transcriptome analysis

The tissue distribution of opossum *GPB* transcription was determined using an available dataset whole transcriptomes containing 19 adult opossum tissues, foetal tissue and newborn tissue [68]; NCBI accession number PRJNA200320). Adult tissues included adipose tissue, brain, colon, diaphragm, eye, heart, intestine, kidney, liver, lung, ovary, pancreas, skeletal muscle, skin (collected from ear pinna), spleen, stomach, teste, thyroid and tongue. Sequences were generated and assembled using normalised libraries as described by Douglas et al. [53]. The tissue distribution of the platypus GBP was determined using a publicly available transcriptome dataset containing sequences from 10 mammalian species originally published by Brawand et al. (accession number PRJNA143627) [60]. The platypus-specific tissues in Brawand et al. included brain, cerebellum, heart, kidney and liver from both male and female animals and testes. Available genomic sequences from both platypus and echidna genomes were then used in a BLASTN analysis in a local database [69]. Initial BLAST was performed with an e-value cut-off e^−4^ to attempt to capture a broad range of GBP transcripts, resulting in a total of 562 transcripts identified. Only those transcripts (36 in total or 16%) that covered 80% or greater of the query were used in further analyses. The identity of these 36 was further confirmed by BLAST to identify the GBP loci sequence in the platypus genome. Accession numbers for platypus and echidna GBP sequences used for the initial BLAST in the local database are as follows: platypus *GBP1/2/3/5*, XM_029064159, XM_029063340, XM_029063338; echidna *GBP1/2/3/5*, XM_038744990; platypus *GBP4/6/7*, XM_039911878, *echidna GBP4/6/7*, XM_038745171, platypus *GBP8*, XM_029066082; echidna *GBP8*, XM_038767322; platypus *GBP9*, XM_029066673; and echidna *GBP9* XM_038767269.

## Supplementary Information

Additional file 1: Table S1. Genbank accession number of the Monotreme and Marsupial sequences included in this study and proposed new nomenclature for Gbp genes. The presence/absence of *GBP* characteristic motifs is also given. Table S2. Log-likelihood scores and statistical tests for alternative phylogenetic hypotheses regarding the placement of GBP8 and GBP9. Three topologies were tested: Hypothesis A (where GBP9 and GBP8 are progressively nested within the GBP1–7 clade); Hypothesis B (GBP8 and GBP9 are nested within the broader GBP1–7 clade) and Hypothesis C (GBP8 and GBP9 form a sister clade to GBP1–7). Outgroup sequences were constrained to form a basal monophyletic clade in all comparisons. Statistical tests were performed using IQ-TREE3 with 10,000 RELL replicates. Plus signs denote the 95% confidence sets. Minus signs denote significant exclusion.

Additional file 2: Data 1. Alignment of the mammals' GBP amino acid sequences used in this study.

Additional file 3: Figure S1. *GBP8 and GBP9* synteny in Mammals. Organisation of the *GBP8* and *GBP9* syntenic regions of marsupials and placentals’ according to genomes available in NCBI (www.ncbi.nlm.nih.org). The diagram is not drawn to scale. Chromosomes are indicated. Double slashes indicate a greater gap/presence of other genetic elements between the represented genes.

Additional file 4: Data 2. Alignment of the mammals' GBP nucleotide sequences used in this study.

## Data Availability

All data analysed during this study are included in this published article [and its supplementary information files].

## Abbreviations

GBPs: Guanylate-binding proteins
GTPases: Guanosine triphosphatases
IFN: Interferon
ISGs: IFN-stimulated genes
Mya: Million years ago
GTP: Guanosine triphosphate
ML: Maximum Likelihood
SH: Shimodaira–Hasegawa
AU: Approximately Unbiased
BLAST: Basic Local Alignment Search Tool
