# Supplementary material for: Deciphering the origins of guanylate-binding proteins in mammals (Monotreme, Marsupials and Placentals)
Source: BMC Biol. 2025 Oct 1;23:292. doi: 10.1186/s12915-025-02403-8 (PMC12486975; doi:10.1186/s12915-025-02403-8)
Supplement: Supplementary file 1 — Additional file 1: Table S1. Genbank accession number of the Monotreme and Marsupial sequences included in this study and proposed new nomenclature for Gbp genes. The presence/absence of GBP characteristic motifs is also given. Table S2. Log-likelihood scores and statistical tests for alternative phylogenetic hypotheses regarding the placement of GBP8 and GBP9. Three topologies were tested: Hypothesis A (where GBP9 and GBP8 are progressively nested within the GBP1–7 clade); Hypothesis B (GBP8 and GBP9 are nested within the broader GBP1–7 clade) and Hypothesis C (GBP8 and GBP9 form a sister clade to GBP1–7). Outgroup sequences were constrained to form a basal monophyletic clade in all comparisons. Statistical tests were performed using IQ-TREE3 with 10,000 RELL replicates. Plus signs denote the 95% confidence sets. Minus signs denote significant exclusion. [file 12915_2025_2403_MOESM1_ESM.docx]

Additional file 1

**Table S1.** Genbank accession number of the Monotreme and Marsupial sequences included in this study and proposed new nomenclature for Gbp genes. The presence/absence of *GBP* characteristic motifs is also given.

| **Accession number** | ***Species*** | **Gene name** | **Proposed classification** | **CaaX motif** | **GxxxxGK (S/T) motif** | **TLRD/TVRD motif** |
| --- | --- | --- | --- | --- | --- | --- |
| XM_001377836 | *Monodelphis domestica* | *GBP1* | *GBP1/2/3/5 a* | *CVII* | Yes | *TLRD* |
| XM_036757824 | *Trichosurus vulpecula* | *GBP1* | *GBP1/2/3/5 a* | *CIIL* | Yes | *TVRD* |
| XM_027877103 | *Vombatus ursinus* | *GBP1* | *GBP1/2/3/5 a* | No | Yes | *TVRD* |
| XM_020988774 | *Phascolarctos cinereus* | *GBP1* | *GBP1/2/3/5 a* | *CIIL* | Yes | *TVRD* |
| XM_023501635 | *Sarcophilus harrisii* | *GBP1* | *GBP1/2/3/5 a* | *CVIS* | Yes | *TVRD* |
| XM_044674139 | *Gracilinanus agilis* | *GBP1* | *GBP1/2/3/5 a* | *CIIS* | Yes | *TLRD* |
| XM_043963142 | *Dromiciops gliroides* | *GBP1* | *GBP1/2/3/5 a* | *CIIC* | Yes | *TVRD* |
| XM_044000619 | *Dromiciops gliroides* | *GBP1* | *GBP1/2/3/5 a* | *CVIC* | Yes | *TVRD* |
| XM_044000624 | *Dromiciops gliroides* | *GBP1* | *GBP1/2/3/5 a* | *CIIC* | Yes | *TVRD* |
| XM_016429840 | *Monodelphis domestica* | *GBP1* | *GBP1/2/3/5 b* | No | Yes | *TLRD* |
| XM_036757829 | *Trichosurus vulpecula* | *GBP1* | *GBP1/2/3/5 b* | No | Yes | *TVRD* |
| XM_027874648 | *Vombatus ursinus* | *GBP1* | *GBP1/2/3/5 b* | No | Yes | *TVRD* |
| XM_044674140 | *Gracilinanus agilis* | *GBP1* | *GBP1/2/3/5 b* | No | Yes | *TLRD* |
| XM_016429837 | *Monodelphis domestica* | *GBP1* | *GBP1/2/3/5 c* | *CIIL* | Yes | *TVRD* |
| XM_036757826 | *Trichosurus vulpecula* | *GBP1* | *GBP1/2/3/5 c* | *CIIS* | Yes | *TVRD* |
| XM_027838227 | *Vombatus ursinus* | *GBP1* | *GBP1/2/3/5 c* | No | Yes | *TVRD* |
| XM_027842115 | *Vombatus ursinus* | *GBP1* | *GBP1/2/3/5 c* | *CIIL* | Yes | *TVRD* |
| XM_020988775 | *Phascolarctos cinereus* | *GBP1* | *GBP1/2/3/5 c* | *CIIL* | Yes | *TVRD* |
| XM_031969369 | *Sarcophilus harrisii* | *GBP1* | *GBP1/2/3/5 c* | *CTIL* | Yes | *TVRD* |
| XM_031969368 | *Sarcophilus harrisii* | *GBP1* | *GBP1/2/3/5 c* | *CTIS* | Yes | *TVRD* |
| XM_031969367 | *Sarcophilus harrisii* | *GBP1* | *GBP1/2/3/5 c* | *CPIL* | Yes | *TVRD* |
| XM_044672240 | *Gracilinanus agilis* | *GBP1* | *GBP1/2/3/5 c* | *CIIL* | No | *TVRD* |
| XM_044000625 | *Dromiciops gliroides* | *GBP1* | *GBP1/2/3/5 c* | *CIIL* | Yes | *TVRD* |
| XM_044000616 | *Dromiciops gliroides* | *GBP1* | *GBP1/2/3/5 c* | No | Yes | *TVRD* |
| XM_016429836 | *Monodelphis domestica* | *GBP6* | *GBP4/6/7* | No | Yes | *TVRD* |
| XM_007480365 | *Monodelphis domestica* | *GBP7* | *GBP4/6/7* | No | Yes | *TVRD* |
| XM_007480361 | *Monodelphis domestica* | *GBP4* | *GBP4/6/7* | No | Yes | *IVRD* |
| XM_007480376 | *Monodelphis domestica* | *GBP4* | *GBP4/6/7* | No | Yes | *No* |
| XM_036757083 | *Trichosurus vulpecula* | *GBP4* | *GBP4/6/7* | No | Yes | *TVRD* |
| XM_036757823 | *Trichosurus vulpecula* | *GBP4* | *GBP4/6/7* | No | Yes | *TVRD* |
| XM_036757820 | *Trichosurus vulpecula* | *GBP6* | *GBP4/6/7* | No | Yes | *TVRD* |
| XM_027838179 | *Vombatus ursinus* | *GBP6* | *GBP4/6/7* | No | Yes | *TVRD* |
| XM_027838077 | *Vombatus ursinus* | *GBP6* | *GBP4/6/7* | No | Yes | *TVRD* |
| XM_027874641 | *Vombatus ursinus* | *GBP4* | *GBP4/6/7* | No | No | *TVRD* |
| XM_020988772 | *Phascolarctos cinereus* | *GBP4* | *GBP4/6/7* | No | Yes | *TVRD* |
| XM_020988773 | *Phascolarctos cinereus* | *GBP7* | *GBP4/6/7* | No | Yes | *TVRD* |
| XM_031969361 | *Sarcophilus harrisii* | *GBP4* | *GBP4/6/7* | No | Yes | *VVRD* |
| XM_031968566 | *Sarcophilus harrisii* | *GBP4* | *GBP4/6/7* | No | Yes | *No* |
| XM_031969370 | *Sarcophilus harrisii* | *GBP4* | *GBP4/6/7* | No | Yes | *AVRD* |
| XM_031969366 | *Sarcophilus harrisii* | *GBP6* | *GBP4/6/7* | No | Yes | *TVRD* |
| XM_031969371 | *Sarcophilus harrisii* | *GBP4* | *GBP4/6/7* | No | No | *No* |
| XM_031969363 | *Sarcophilus harrisii* | *GBP4* | *GBP4/6/7* | No | Yes | *TVRD* |
| XM_044672236 | *Gracilinanus agilis* | *GBP4* | *GBP4/6/7* | No | Yes | *TVRD* |
| XM_044672238 | *Gracilinanus agilis* | *GBP6* | *GBP4/6/7* | No | Yes | *TVRD* |
| XM_044674842 | *Gracilinanus agilis* | *GBP6* | *GBP4/6/7* | No | Yes | *TVRD* |
| XM_044672239 | *Gracilinanus agilis* | *GBP7* | *GBP4/6/7* | No | Yes | *TVRD* |
| XM_044000611 | *Dromiciops gliroides* | *GBP4* | *GBP4/6/7* | No | Yes | *TVRD* |
| XM_044000609 | *Dromiciops gliroides* | *GBP4* | *GBP4/6/7* | No | Yes | *TVRD* |
| XM_044000615 | *Dromiciops gliroides* | *GBP4* | *GBP4/6/7* | No | Yes | *TVRD* |
| XM_044003719 | *Dromiciops gliroides* | *GBP4* | *GBP4/6/7* | No | Yes | *TVRD* |
| XM_044004733 | *Dromiciops gliroides* | *GBP6* | *GBP4/6/7* | No | Yes | *TVRD* |
| XM_029064159 | *Ornithorhynchus anatinus* | *GBP1* | *GBP1/2/3/5* | *CVIL* | Yes | *TVRD* |
| XM_029063340 | *Ornithorhynchus anatinus* | *GBP1* | *GBP1/2/3/5* | *CTIL* | Yes | *TVRD* |
| XM_029063338 | *Ornithorhynchus anatinus* | *GBP1* | *GBP1/2/3/5* | *CIII* | Yes | *TVRD* |
| XM_038745030 | *Tachyglossus aculeatus* | *GBP1* | *GBP1/2/3/5* | *CVIL* | Yes | *TVRD* |
| XM_038744990 | *Tachyglossus aculeatus* | *GBP1* | *GBP1/2/3/5* | *CIII* | Yes | *TVRD* |
| XM_029066082 | *Ornithorhynchus anatinus* | *GBP1* | *GBP8* | *CLIQ* | Yes | *AVRD* |
| XM_038767322 | *Tachyglossus aculeatus* | *GBP1* | *GBP8* | *CPIQ* | Yes | *VVRD* |
| XM_039911878 | *Ornithorhynchus anatinus* | *GBP4* | *GBP4/6/7* | No | Yes | *AVRD* |
| XM_038745171 | *Tachyglossus aculeatus* | *GBP4* | *GBP4/6/7* | No | Yes | *AVRD* |
| XM_038767269 | *Tachyglossus aculeatus* | *GBP1* | *GBP9* | No | Yes | *TVRD* |
| XM_029066673 | *Ornithorhynchus anatinus* | *GBP1* | *GBP9* | No | Yes | *TVRD* |

**Table S2.** Log-likelihood scores and statistical tests for alternative phylogenetic hypotheses regarding the placement of GBP8 and GBP9. Three topologies were tested: Hypothesis A (where GBP9 and GBP8 are progressively nested within the GBP1–7 clade); Hypothesis B (GBP8 and GBP9 are nested within the broader GBP1–7 clade) and Hypothesis C (GBP8 and GBP9 form a sister clade to GBP1–7). Outgroup sequences were constrained to form a basal monophyletic clade in all comparisons. Statistical tests were performed using IQ-TREE3 with 10,000 RELL replicates. Plus signs denote the 95% confidence sets. Minus signs denote significant exclusion.

| **Tree Hypothesis** | **log L** | **delta L** | **bp-RELL** | **p-KH** | **p-SH** | **p-WKH** | **p-WSH** | **c-ELW** | **p-AU** |
| --- | --- | --- | --- | --- | --- | --- | --- | --- | --- |
| Hypothesis A | - 185938 | 9.4744 | 0.341+ | 0.383+ | 0.432+ | 0.384+ | 0.430+ | 0.341+ | 0.374+ |
| Hypothesis B | - 185929 | 0 | 0.364+ | 0.503+ | 1+ | 0.503+ | 0.703+ | 0.364+ | 0.534+ |
| Hypothesis C | - 185929 | 0.12697 | 0.295+ | 0.497+ | 0.756+ | 0.497+ | 0.754+ | 0.296+ | 0.555+ |
