# Supplementary figures and images for: Deciphering the origins of guanylate-binding proteins in mammals (Monotreme, Marsupials and Placentals)
Source: BMC Biol. 2025 Oct 1;23:292. doi: 10.1186/s12915-025-02403-8 (PMC12486975; doi:10.1186/s12915-025-02403-8)

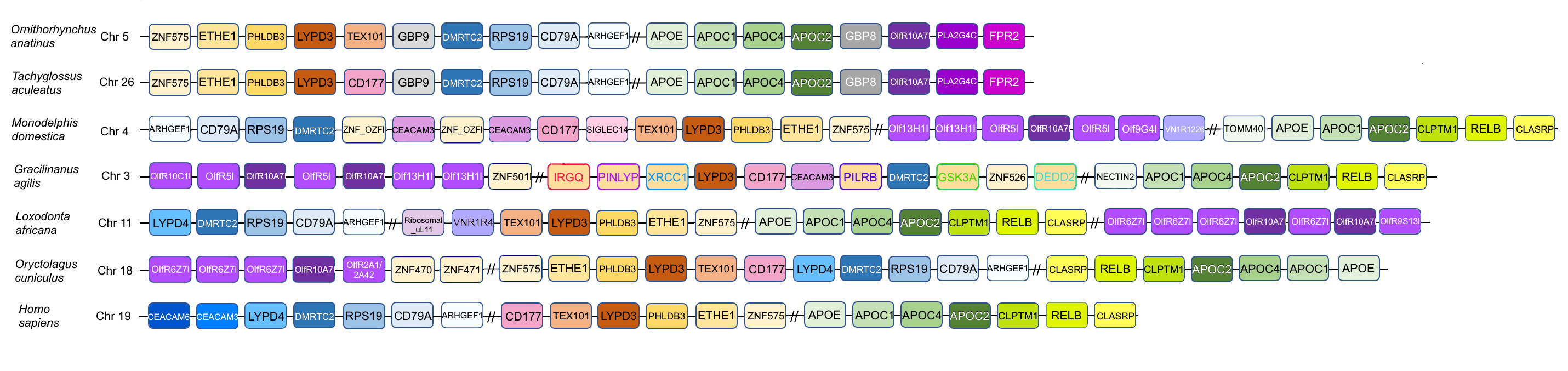

Supplement: Supplementary file 3 — Additional file 3: Figure S1. GBP8 and GBP9 synteny in Mammals. Organisation of the GBP8 and GBP9 syntenic regions of marsupials and placentals’ according to genomes available in NCBI (www.ncbi.nlm.nih.org). The diagram is not drawn to scale. Chromosomes are indicated. Double slashes indicate a greater gap/presence of other genetic elements between the represented genes. [file 12915_2025_2403_MOESM3_ESM.tiff]
